# Retraction Note: Eficacy of pentoxifylline treatment for neonatal sepsis: a meta-analysis of randomized controlled studies

**DOI:** 10.1186/s13052-021-00979-9

**Published:** 2021-02-12

**Authors:** Jun Tian, Peifang Shen, Kaiyu Pan, Qiong Zhou

**Affiliations:** 1 Department of pediatrics, The First people’s Hospital of Xiaosha, Hangzhou, China; 2 Department of pediatrics, Children’s Hospital of Hangzhou, No 318 Chaowang Road, Hangzhou, 310005 Zhejiang Province People’s Republic of China

**Retraction Note: Ital J Pediatr 45, 108 (2019)**

**https://doi.org/10.1186/s13052-019-0697-8**

The Editor-in-Chief has retracted this article [1] because it contains material that substantially overlaps with an article by Peiyun Peng and Yunfeng Xia [2]. All authors agree to this retraction.
